# Porcine β-Defensin 2 Expressed in *Pichia pastoris* Alleviates Enterotoxigenic *Escherichia coli*-Induced Intestinal Injury and Inflammatory Response in Mice

**DOI:** 10.3390/ani15101389

**Published:** 2025-05-11

**Authors:** Shuaiyang Wang, Huaixia Li, Yaxue Huang, Wenxiao Zhuo, Tingting Li, Tingting Jiang, Qi Huang, Rui Zhou

**Affiliations:** 1 National Key Laboratory of Agricultural Microbiology, College of Veterinary Medicine, Huazhong Agricultural University, Wuhan 430070, China; wshuaiyang@webmail.hzau.edu.cn (S.W.); hx_lee@webmail.hzau.edu.cn (H.L.); yaxuehuang@webmail.hzau.edu.cn (Y.H.); zhuowenxiao@webmail.hzau.edu.cn (W.Z.); li.tingting@webmail.hzau.edu.cn (T.L.); jtt@webmail.hzau.edu.cn (T.J.); qhuang@mail.hzau.edu.cn (Q.H.); 2 International Research Center for Animal Disease, Ministry of Science & Technology of China, Wuhan 430070, China; 3 The Cooperative Innovation Center of Sustainable Pig Production, Wuhan 430070, China

**Keywords:** porcine β-defensin 2, *Pichia pastoris*, enterotoxigenic *Escherichia coli*, antimicrobial activity, mouse infection model, intestinal morphology, pro-inflammatory cytokines

## Abstract

Enterotoxigenic *Escherichia coli* (ETEC), a common intestinal pathogen, can colonize the intestines and induce diarrhea in piglets, which brings great economic losses to the swine industry. Antibiotics are recommended to the treatment for diarrhea caused by ETEC in weaned piglets. However, with the emergence and spread of multidrug-resistant ETEC, there is an urgent need to develop alternatives to antibiotics. Due to the unique antibacterial mechanism of targeting bacterial membranes, antimicrobial peptides (AMPs) are promising candidates. In this study, the activity of crude recombinant porcine β-defensin 2 (rPBD2) expressed in *Pichia pastoris* (*P. pastoris*) was measured in vitro. Mice infected with ETEC were orally administered 16, 8, and 4 AU crude rPBD2 for 7 consecutive days to evaluate its anti-infective activity in vivo. The results showed that in addition to broad antibacterial activity against Gram-positive and -negative bacteria, crude rPBD2 displayed high tolerance to temperatures ranging from 20 to 60 °C, a broad range of pH, trypsin, pepsin, and physiological concentrations of salts. In an ETEC-induced mouse model, the oral administration of crude rPBD2 decreased diarrhea scores and the intestinal/carcass ratio and alleviated body weight loss. Additionally, crude rPBD2 decreased bacterial loads in stools and the colon (HP group), and the levels of serum pro-inflammatory cytokines IL-6 (HP group) and TNF-α (HP and MP groups), and increased the villus height and the ratio of villus height to crypt depth (VH/CD) in the ileum (HP and MP groups). Our study provides a cost-effective way for PBD2 production and identifies it as a promising candidate to combat ETEC-induced infection.

## Simple Summary

Porcine β-defensin 2 enhances immunity and protects the host from bacterial infection. In this study, we evaluated the in vitro antibacterial activity of crude recombinant porcine β-defensin 2 expressed in *Pichia pastoris* and the in vivo antibacterial activity in an enterotoxigenic *Escherichia coli*-induced mouse model. The results indicated that crude recombinant porcine β-defensin 2 had broad-spectrum antibacterial activity against Gram-positive and -negative bacteria. Crude recombinant porcine β-defensin 2 also showed high resistance to pH, proteases, salts, and temperatures ranging from 20 to 60 °C. In addition, the oral administration of crude recombinant porcine β-defensin 2 alleviated clinical symptoms, intestinal damage, and inflammatory response and decreased pathogen loads in stools and the colon. Our results indicate that porcine β-defensin 2 expressed in *Pichia pastoris* is an attractive alternative to traditional antibiotics that can be used to combat enterotoxigenic *Escherichia coli*-induced infection.

## 1. Introduction

Pathogenic *Escherichia coli* (*E. coli*) is a common pathogen that causes intestinal diseases in animals and humans [1]. Enterotoxigenic *E. coli* (ETEC) is one of the predominant intestinal pathogenic *E. coli* causing diarrhea in children and young animals [2,3,4]. In addition, ETEC is the main reason for postweaning diarrhea (PWD) in pigs [4,5,6,7]. Piglets infected with ETEC are characterized by watery feces, growth retardation, and high mortality rate, which induces great economic losses to the swine industry [6,8,9]. ETEC mainly expresses two kinds of virulence factors: adhesins or fimbriae and enterotoxins [6]. Once ingested orally by piglets, ETEC expresses fimbriae to adhere to specific receptors present in intestinal epithelial cells [9]. Then, ETEC secretes two enterotoxins, heat-stable toxins (STs) and heat-labile toxins (LTs), that can destroy intestinal barrier function, induce inflammatory response, and disrupt the balance of intestinal microbiota, ultimately leading to intestinal diseases [10,11,12].

Antibiotics are the common treatment to antagonize bacterial infections and are also widely used in pig production to combat ETEC infection [6,13]. However, the long-term use of antibiotics has caused antibiotic resistance and antibiotic residues in livestock, which has resulted in a significant threat to global public health [14,15,16]. Moreover, low concentrations of antibiotics promote the emergence and spread of bacteria of antibiotic resistance and induce the formation of biofilm [17,18,19]. Therefore, it is urgent to develop alternative therapies to traditional antibiotics. Antimicrobial peptides (AMPs) are plausible candidates for the prevention and treatment of bacterial infections [20]. The aim of this study is to test the antibacterial infection efficacy of an AMP (β-defensin) in an ETEC K88 mouse model.

AMPs, also known as host defense peptides (HDPs), are amphipathic and cationic small molecules, comprising 10–100 amino acids, and play an important role in innate immune system [21,22]. AMPs are widespread in animals, plants, and microbes, possessing antibacterial, anti-viral, anti-fungal, anti-parasitic, and anti-cancer activities, as well as immune regulatory functions [20,23]. In addition, most AMPs exert antibacterial activity by targeting the cell membranes of bacteria. Because the membrane structures of bacteria are relatively stable, it is not easy to develop resistance to AMPs [24,25]. Due to their unique antibacterial mechanism compared to traditional antibiotics, AMPs have been used to treat ETEC infection. For instance, lasso peptide microcin J25 (MccJ25) attenuated ETEC-induced intestinal barrier dysfunction by increasing the tight junction protein expression of the small intestine and helped improve host health in a mouse model [10]. Cecropin AD attenuated piglet diarrhea caused by ETEC and enhanced performance by increasing immune status and nitrogen and energy retention [26]. Additionally, antimicrobial peptide KR-32 improved the growth performance, fatty acid absorption, and intestinal morphology of ETEC K88-infected piglets [27].

Defensins, an important family of AMPs, play a crucial role in pathogen antagonization, intestinal barrier maintenance, and immunomodulation in mammals [28,29]. According to their structural characteristics, mammalian defensins are divided into three subfamilies: α-, β-, and θ-defensin [30]. In pigs, only the β-defensin subfamily has been identified so far [31]. Porcine β-defensin 2 (PBD2) is widely distributed in the tongue, liver, kidney, small intestine, and large intestine of pigs [32], and is the most extensively investigated member among all identified β-defensins in pigs [31]. PBD2 shows great antibacterial activity against Gram-positive and -negative bacteria [33,34]. In addition, synthetic PBD2 can inhibit the proliferation of pseudorabies virus (PRV) as well as porcine reproductive and respiratory syndrome virus (PRRSV) in vitro [33,35]. The oral administration of synthetic PBD2 improved growth performance, reduced inflammatory cytokine levels, and affected intestinal morphological indices in weaned piglets infected with ETEC [36], and synthetic PBD2 also attenuated inflammation and mucosal lesions in dextran sodium sulfate (DSS)-induced colitis mice by inhibiting the NF-κB signaling pathway [37]. Dietary supplementation with crude recombinant PBD2, which was expressed in *Pichia pastoris* (*P. pastoris*), improved growth performance and reduced the incidence of diarrhea in weaned piglets [38]. In another study, His-tagged PBD2, which was expressed in *E. coli*, attenuated the inflammatory response induced by *E. coli* in IPEC-J2 cells via inhibiting the TLRs-TAK1-NF-κB/MAPK signaling pathway [39,40]. In our previous studies, PBD2-overexpressing transgenic mice had enhanced resistance to PRV and *Salmonella* [35,41], and PBD2-overexpressing transgenic pigs had enhanced resistance to *Actinobacillus pleuropneumoniae* (*A. pleuropneumoniae*), *Glasserella parasuis* (*G. parasuis*), *Streptococcus suis* (*S. suis*), and swine influenza virus (SIV) [31,42,43,44]. These previous studies on PBD2 make it a plausible antibiotic substitute. However, the high cost of chemical synthesis has seriously hindered the application of AMPs in animal husbandry [45]. Heterologous expression is the most economic and efficient method for the large-scale production of AMPs [46]. In our previous study, the oral administration of synthetic PBD2 can alleviate *S. Typhimurium*-induced inflammation in mice [41]. It is unknown whether PBD2 expressed in *P. pastoris* has antibacterial activity against ETEC-induced infection in vivo. In this study, PBD2 was expressed in *P. pastoris* in which the induction conditions, including the methanol concentrations and induction time, were optimized. The antibacterial spectrum and stability of the crude recombinant PBD2 (rPBD2) were measured, and its in vivo therapeutic efficacy was determined in an ETEC K88-infected mouse model by oral administration. This study will provide a useful reference for the use of PBD2 to treat ETEC-induced infection.

## 2. Materials and Methods

### 2.1. Strains and Plasmids

*E. coli* DH5α was purchased from Weidi Bio (Shanghai, China) and was used for the multiplication of the expression plasmid. *P. pastoris* X-33 and pPICZαA vector were used for the secretory expression of rPBD2. *E. coli* strains ATCC 25922, ETEC7, ETEC17, ETEC20, EPEC28, EPEC48, EPEC66, EPEC133, 72, and PCN033, *Salmonella Typhimurium* (*S. typhimurium*) strains ATCC 14028, CVCC 542, and CVCC 212197, *Salmonella pullorum* (*S. pullorum*) C79-13, *Pasteurella multocida* (*P. multocida*) strains 9261 and HB03, *A. pleuropneumoniae* 4074, *Staphylococcus aureus* (*S. aureus*) strains ATCC 29213 and 1213M4A, and *S. suis* strains SC19 and 0810 used for antimicrobial testing are stocked in our laboratory. ETEC7, an ETEC isolate from a diarrheal piglet was used to establish a diarrhea mouse model [47].

### 2.2. Enzymes and Chemicals

The restriction enzymes QuickCut™ *Xho*I, QuickCut™ *Sal*I, and QuickCut™ *Sac*I were supplied by Takara Bio (Beijing, China). The ClonExpress MultiS One Step Cloning Kit was purchased from Vazyme (Nanjing, China). Zeocin was purchased from InvivoGen (San Diego, CA, USA). The plasmid extraction kit and the DNA purification kit were from Tiangen (Beijing, China). The Enzyme-Linked Immunosorbent Assay (ELISA) kits of IL-6, IL-10, and TNF-α were from Multi Sciences (Hangzhou, China). All other chemicals used were of analytical grade.

### 2.3. Construction of PBD2 Expression Plasmid pPPBD2

The cDNA sequence of PBD2 (GenBank accession no. AY506573.1) was optimized for *P. pastoris* (www.kazusa.or.jp/codon/, accessed on 23 October 2022). The optimized sequence was synthesized by Sangon Biotech (Shanghai, China). The DNA fragment containing an *Xho*I restriction site, a Kex2 protease cleavage site, an optimized PBD2 sequence, a stop codon, and a *Sal*I restriction site was amplified by polymerase chain reaction (PCR) using the primer pairs PBD2F and PBD2R. The DNA fragment was purified and then inserted into pPICZαA digested by *Xho*I and *Sal*I. The recombinant expression plasmid was introduced into a DH5α competent cell and selected on LB plates containing 25 μg/mL Zeocin. The correct recombinant expression plasmid pPPBD2 was verified by PCR using the primer set 5′AOX1 and 3′AOX1 and DNA sequencing. The primers used in this study are listed in Table 1.

### 2.4. Transformation and Expression of rPBD2 in P. pastoris

The pPPBD2 plasmid was linearized by *Sac*I and then was transformed into *P. pastoris* X-33 by electroporation according to Invitrogen’s instructions. Positive transformants were selected on YPD (Solarbio, Beijing, China) plates containing 100 μg/mL Zeocin and were then further verified by PCR using the primers 5′AOX1/3′AOX1. Positive transformants were inoculated into 5 mL YPD and cultured overnight under the conditions of 29 °C and 200 rpm. The cultures were transferred to 25 mL BMGY (Solarbio, Beijing, China) medium and cultured under the same conditions until the OD_600_ reached 5.0. Then, cultures were harvested by centrifugation and pellets were resuspended in 50 mL BMMY (Solarbio, Beijing, China) medium to an OD_600_ of 1.0. Methanol was added every 24 h to a final concentration of 1.0% (*v*/*v*) to induce the expression of rPBD2. The supernatant was collected by centrifugation after 72 h induction. The expression of rPBD2 was determined by antimicrobial activity against *S. aureus* ATCC 29213 and Tricine sodium dodecyl sulphate–polyacrylamide gel electrophoresis (Tricine SDS-PAGE) [48]. *P. pastoris* X-33 harboring pPICZαA was used as a negative control. To enhance the expression levels of rPBD2 in *P. pastoris* X-33, the induction conditions were also optimized at different induction times (24, 48, 72, 96, 120, and 144 h) and methanol concentrations (0.5%, 1.0%, 1.5%, 2.0%, and 2.5%). All experiments were performed in biological triplicate.

### 2.5. Antimicrobial Titer Assay

The activity units of rPBD2 supernatant after 120 h induction with 1% methanol at 29 °C and 200 rpm were determined as previous described with modifications [49]. The rPBD2 supernatant was continuously diluted twofold, and 100 μL supernatant was co-incubated with 100 μL *S. aureus* ATCC 29213 (~10^6^ CFU/mL). The titer was defined as the reciprocal of the highest dilution that killed 99% bacteria. Thus, the activity unit (AU) of rPBD2 supernatant mL^−1^ was defined as 2^n^ × 1000 μL/100 μL.

### 2.6. Antibacterial Spectrum Assay

The OD_600_ of bacteria in logarithmic growth stage was adjusted to 0.02, and 100 μL cell suspension was incubated with 100 μL rPBD2 supernatant (10 AU/mL) for 8 h at 37 °C without shaking. Then, the OD_600_ was measured, and the inhibition rate was calculated as ((OD_600 (Positive)_ − OD_600 (rPBD2)_)/OD_600 (Positive)_) × 100%, where OD_600 (Positive)_ is the average OD_600_ value of bacteria without rPBD2 supernatant treatment, and OD_600 (rPBD2)_ is the average OD_600_ value of bacteria with rPBD2 supernatant treatment for 8 h.

### 2.7. Stability Assay of Crude rPBD2

The rPBD2 supernatant was incubated at 20, 40, 60, 80, and 100 °C for 30 min to test thermal stability. The pH value of the rPBD2 supernatant was adjusted to 2, 4, 6, 8, and 10, and then it was incubated for 2 h at 37 °C to test pH stability. The rPBD2 supernatant was incubated with trypsin, pepsin, and proteinase K at 37 °C for 2 h to measure protease stability, and the final concentration of proteases was 100 μg/mL. To test salt stability and serum stability, *S. aureus* ATCC 29213 was incubated with rPBD2 supernatant supplemented with 150 mM NaCl, 4.5 mM KCl, 6 μM NH_4_Cl, 8 μM ZnCl_2_, 1 mM MgCl_2_, 2.5 mM CaCl_2_, 4 μM FeCl_3_, and 5% or 10% fetal bovine serum (FBS) for 8 h, respectively. The remaining antimicrobial activity of the rPBD2 supernatant was determined as described in Section 2.7.

### 2.8. Animal Experiment Design

The animal experiment was approved by the Animal Ethics and Welfare Committee of Huazhong Agricultural University, Wuhan, China (HZAUMO-2023-0264). The animal model was established as in our previous experiment [50]. Female ICR mice (3–4 weeks old) were obtained from the Laboratory Animals Center of Huazhong Agricultural University. After 5 days of adaptation, 36 mice were randomly assigned into 6 groups (n = 6/group), namely, a control group (only phosphate-buffered saline (PBS) treatment), the ETEC7 group (ETEC7 and PBS treatment), the COL group (ETEC7 and 9000 U colistin sulfate treatment), the HP group (ETEC7 and 16 AU rPBD2 treatment), the MP group (ETEC7 and 8 AU rPBD2 treatment), and the LP group (ETEC7 and 4 AU rPBD2 treatment). The mice were fed antibiotics, including kanamycin (400 mg/L), gentamicin (35 mg/L), vancomycin (45 mg/L), metronidazole (215 mg/L), and colistin (850 U/mL), in drinking water for 72 h and were then fed normal water for another 24 h. All mice were fasted for 12 h and then were orally gavaged with 200 μL 3% NaHCO_3_ 30 min prior to the challenge with 10^9^ CFU of ETEC7. The mice were orally infected with ETEC7 once a day for 3 days. After 6 h ETEC7 infection, mice in the COL group were orally gavaged with 9000 U colistin sulfate, while those in the HP, MP, and LP groups were gavaged with 16 AU, 8 AU, and 4 AU crude rPBD2, respectively. The control and ETEC7 groups were administrated with the same volume of PBS. All mice were treated with PBS, colistin sulfate, or rPBD2 for 7 continuous days.

### 2.9. Clinical Symptoms and Sample Collection

Body weight change was calculated as ((final body weight − initial body weight)/initial body weight) × 100%. Diarrhea scores were recorded at 1, 3, 5, and 7 days post infection (dpi) based on the following scoring criteria: (1) normal stool: 0 point; (2) color change/consistency: 1 point; (3) presence of wet tail or mucosa: 2 points; (4) watery stool: 3 points. A score of 1 was considered diarrhea [51]. Fresh stools and 1 cm colon were collected and suspended in sterile PBS. Ten-fold serial dilutions of stool or colon homogenate were plated on MacConkey plates containing 100 μg/mL ampicillin. At 7 dpi, all mice were euthanized and blood was collected and centrifuged for 10 min at 1000× *g* and 4 °C. The serum was collected to measure the levels of pro-inflammatory cytokines IL-6, IL-10, and TNF-α using ELISA kits according to the manufacturer’s instructions. Intestine water content (intestine/carcass ratio) was measured as described previously [52]. The cecum index was calculated as cecum weight (g)/body weight (g). Ileum tissues were excised from the mice and fixed in 4% paraformaldehyde for hematoxylin and eosin (HE) staining.

### 2.10. Statistical Analysis

The data that had a normal distribution were analyzed using one-way analysis of variance (ANOVA) with SPSS V20 software; otherwise, a Mann–Whitney U-test was performed. The results were represented as the mean ± standard error of the mean (SEM). * and ** represent *p* < 0.05 and *p* < 0.01, respectively.

## 3. Results

### 3.1. Construction of rPBD2 Expression Plasmid and Screening of Positive P. pastoris Transformants

**Figure 1:**
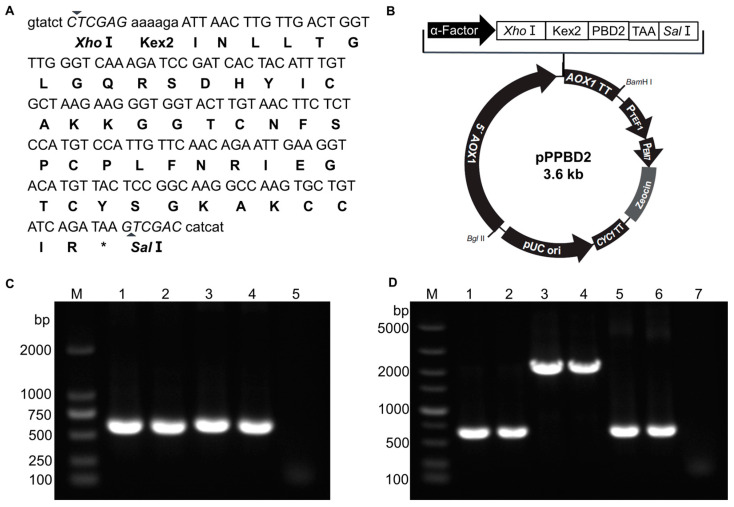
Construction of the PBD2 expression plasmid and its *P. pastoris* transformants. (**A**) Codon-optimized cDNA sequence of PBD2 for *P. pastoris* (upper line) and its corresponding amino acid sequence (lower line). *, stop codon. (**B**) Schematic diagram of recombinant plasmid pPPBD2. (**C**) PCR identification of pPPBD2. M, Marker; 1–2, plasmid pPICZαA; 3–4, pPPBD2; 5, negative control. (**D**) Identification of *P. pastoris* transformants that have the highest inhibition rate against ATCC 29213. M, Marker; 1–2, positive transformants; 3–4, *P. pastoris* X33; 5–6, pPPBD2 plasmid; 7, negative control.

The cDNA sequence of PBD2 was optimized to ensure PBD2 was efficiently expressed in *P. pastoris* (Figure 1A). The PBD2 cDNA was amplified from plasmid pUC57-PBD2 by PCR and then inserted into *Xho*I- and *Sal*I-digested pPICZαA by homologous recombination using the ClonExpress MultiS One Step Cloning Kit (Figure 1B). Correct recombination plasmid pPPBD2 was obtained after PCR verification (Figure 1C) and DNA sequencing. The recombinant plasmid pPPBD2 was linearized by restriction endonuclease *Sac*I and was then transformed into competent cells of *P. pastoris* X-33 by electroporation. *P. pastoris* positive transformants were selected on YPD plates containing 100 μg/mL Zeocin. We obtained sixty positive transformants after PCR verification using primer pairs 5′AOX1 and 3′AOX1.

### 3.2. Screening of High-Expression Strain for rPBD2

**Figure 2:**
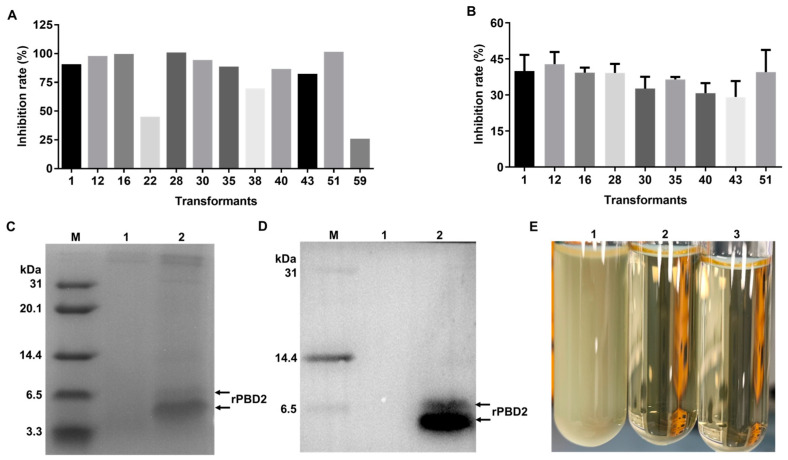
Expression of PBD2 in *P. pastoris.* (**A**) Preliminary screening of positive transformants. In total, 100 μL supernatant of different transformants was co-incubated with 100 μL *S. aureus* ATCC 29213 with an OD_600_ of 0.02, and the OD_600_ was measured after 8 h. Inhibition ratios greater than 15% are shown. (**B**) Secondary screening of positive transformants. In total, 500 μL supernatant was co-incubated with 500 μL ATCC 29213 (5 × 10^5^ CFU/mL) for 1 h at 37 °C without shaking, and 100 μL from each sample was withdrawn to count the bacterial number. (**C**) Tricine SDS-PAGE analysis of rPBD2-51 with 1% methanol induction for 72 h. M, Marker; 1, 15 μL supernatant from the transformant which harbors the pPICZαA vector; 2, 15 μL supernatant from rPBD2-51. (**D**) Western blot analysis of rPBD2-51 using PBD2 monoclonal antibody. M, Marker; 1, pPICZαA vector supernatant; 2, rPBD2-51 supernatant. (**E**) The rPBD2 supernatant inhibits the growth of ATCC 29213. 1, The supernatant from the transformant which harbors the pPICZαA vector; 2, The supernatant from rPBD2-51; 3, Ampicillin.

The sixty positive transformants were induced by 1% methanol for 72 h and then the supernatants were collected to test the antibacterial activity. Twelve transformants had inhibition ratios greater than 15% (Figure 2A) and the others had lower inhibition ratios or no antibacterial activity against the tested strain. Nine of the twelve transformants (rPBD2-1, -12, -16, -28, -30, -35, -40, -43, and -51) were further tested (Figure 2B). After 1 h incubation at 37 °C without shaking, rPBD2-51 was selected for subsequent experiments due to the relatively high antibacterial activity of its culture supernatant. The rPBD2-51 transformant was verified by PCR and DNA sequencing again. The PCR product was consistent with the expected size (Figure 1D) and the correct DNA sequence.

To further verify the expression of rPBD2 in *P. pastoris*, Tricine SDS-PAGE and Western blot analysis was performed. It revealed that two clear bands could be observed around 6.5 kDa from the supernatant of rPBD2-51, whereas no corresponding band was observed from the supernatant of *P. pastoris* X-33 containing pPICZαA empty vector (Figure 2C). The two bands could specifically react with the PBD2 monoclonal antibody [43] (Figure 2D). As expected, strong antibacterial activity was detected for the rPBD2-51 supernatant (Figure 2E). These results indicated that the recombinant *P. pastoris* clone rPBD2-51 can produce biologically active PBD2.

### 3.3. Optimization of Induction Conditions

**Figure 3:**
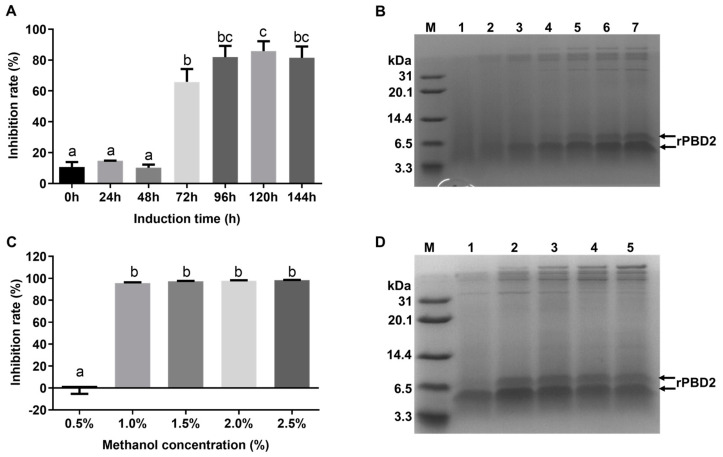
Optimization of induction conditions for rPBD2 production in *P. pastoris*. (**A**,**B**) Optimization of induction times among 0, 24, 48, 72, 96, 120, and 144 h with 1% methanol. (**C**,**D**) Optimization of inducer concentrations among 0.5%, 1%, 1.5%, 2.0%, and 2.5% methanol induced for 144 h. Different superscript lowercase letters within each group indicate significantly different values (*p* < 0.05).

To increase the yield of rPBD2 in recombinant *P. pastoris*, the induction conditions were optimized. When the inducer was set at 1.0% methanol, and the supernatant was collected every 24 h, the antibacterial activity increased with the extension in induction time and reached the maximum after 120 h induction (Figure 3A). Tricine SDS-PAGE analysis also suggested that the rPBD2 expression level increased with the increase in induction time (Figure 3B). The induction concentration of methanol was also optimized. Every 24 h, 0.5%, 1.0%, 1.5%, 2.0%, and 2.5% was added, and the supernatant was collected after 120 h induction. The results showed that the culture supernatant from the culture induced with 1.0%, 1.5%, 2.0%, and 2.5% methanol had similar rPBD2 expression and antimicrobial activity, while induction with 0.5% methanol did not show significant antimicrobial activity (Figure 3C,D). The activity units of the rPBD2 supernatant induced with 1% methanol for 120 h reached 20 AU/mL.

### 3.4. Antimicrobial Spectrum of Crude rPBD2

As shown in Table 2, crude rPBD2 from the culture supernatant of recombinant *P. pastoris* showed a broad spectrum of antibacterial activity against Gram-negative and -positive bacteria. It had an inhibition rate of 83.03–92.16% against *E. coli*, 77.44–89.10% against *Salmonella*, 75.44–78.48% against *P. multocida*, 82.84% against *A. pleuropneumoniae*, 82.51–88.56% against *S. aureus*, and 66.31–77.47% against *S. suis*.

### 3.5. Stability of Crude rPBD2

**Figure 4:**
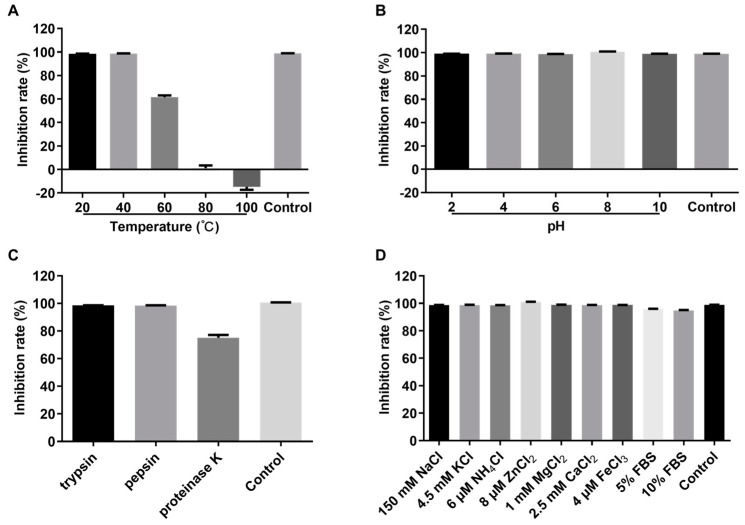
Stability of rPBD2 to temperature, acids, proteases, and salts. (**A**) Effect of temperature on rPBD2 activity against *S. aureus* ATCC 29213. (**B**) Effect of pH on rPBD2 activity against ATCC 29213. (**C**) Effect of proteases on rPBD2 activity against ATCC 29213. (**D**) Effect of physiological concentrations of different salts and fetal bovine serum (FBS) on rPBD2 activity against ATCC 29213.

In order to evaluate the stability of crude rPBD2 in different conditions, the antibacterial activity against *S. aureus* ATCC 29213 was measured. As shown in Figure 4A, the activity of crude rPBD2 after incubation at 20 °C or 40 °C for 30 min was comparable to that of the untreated rPBD2. When incubated at 60 °C, the inhibition rate of rPBD2 decreased to about 60%. When the incubation temperature reached 80 °C, the antibacterial activity was completely abolished. The effect of pH on rPBD2 activity was analyzed. The results showed that a wide range of pH from 2 to 10 had no influence on crude rPBD2 activity (Figure 4B). Moreover, it was shown that crude rPBD2 was resistant to trypsin and pepsin, but the activity was decreased to about 75% after proteinase K treatment, suggesting that rPBD2 was sensitive to proteinase K but not trypsin or pepsin (Figure 4C). Under the treatment of different kinds and concentration of salts, and 5% or 10% fetal bovine serum, crude rPBD2 maintained a high activity (Figure 4D).

### 3.6. Efficacy of Crude rPBD2 on ETEC K88-Infected Mice

**Figure 5:**
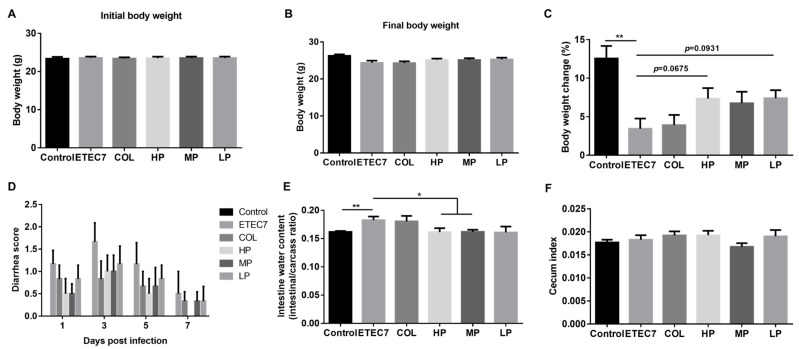
Anti-infective efficacy of rPBD2 in ETEC K88-infected mice. (**A**) Initial body weight before ETEC7 infection. (**B**) Final body weight at 7 dpi. (**C**) Body weight changes up to 7 dpi. (**D**) Diarrhea score at 1, 3, 5, and 7 dpi. (**E**) Intestine water content (intestine/carcass ratio) at 7 dpi. (**F**) Cecum index at 7 dpi. Data are presented as means ± standard error of the mean (SEM). * *p* < 0.05; ** *p* < 0.01.

An ETEC infection mouse model was used to evaluate the in vivo antibacterial activity of crude rPBD2. The mice were infected with ETEC, followed by treatment with different concentrations of crude rPBD. It was shown that following infection, no significant differences were observed on the initial body weight and final body weight among different groups (*p* > 0.05) (Figure 5A,B). However, the body weight changes of ETEC7-infected mice were lower than the control group (*p* < 0.05), and after the oral administration of 16 AU or 4 AU of crude rPBD2, the ETEC7-infected mice had a tendency to attenuated lower body weight changes (*p* = 0.0675 and *p* = 0.0931, respectively) (Figure 5C). Compared with the ETEC7 group, the COL, HP, MP, and LP groups had relative lower diarrhea scores but without significant differences (Figure 5D). However, 16 AU or 8 AU of crude rPBD2 could decrease the intestine water content (intestinal/carcass ratio) (*p* < 0.05) (Figure 5E). There were no significant differences in the cecum index among the groups (Figure 5F).

**Figure 6:**
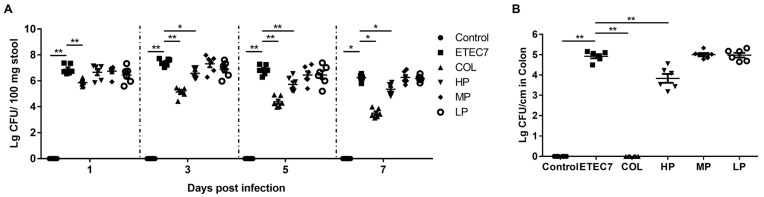
Effect of rPBD2 on *E. coli* loads in stool and colon of ETEC K88-infected mice. (**A**) *E. coli* loads in stool at 1, 3, 5, and 7 dpi. (**B**) *E. coli* loads in colon at 7 dpi. Data are presented as means ± standard error of the mean (SEM). * *p* < 0.05; ** *p* < 0.01.

The bacterial loads of *E. coli* K88 in feces and the colon were determined. As shown in Figure 6A, compared with the ETEC7 group, the bacterial loads in feces in the HP group and the COL group were significantly decreased (*p* < 0.05), whereas no significant differences were observed between the ETEC7 group and the MP group, or between the ETEC7 group and the LP group. In addition, the colonized numbers of *E. coli* K88 in the colon in the HP group and the COL group were also lower than those in the ETEC7 group (*p* < 0.05), while no significant differences were observed between the ETEC7 group and the MP group, or between the ETEC7 group and the LP group (Figure 6B).

**Figure 7:**
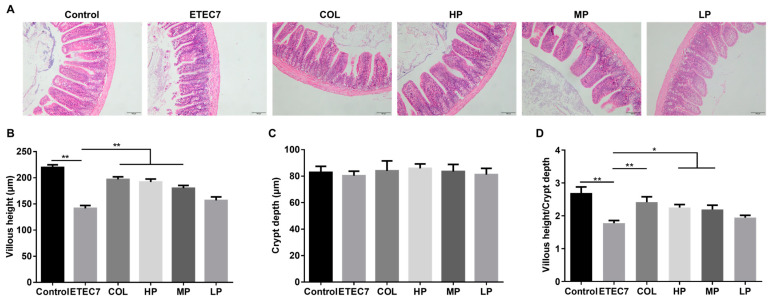
Effect of rPBD2 on the ileum morphology of ETEC K88-infected mice at 7 dpi. (**A**) HE staining of the ileum. The ileum tissues were collected, embedded in paraffin, sectioned, stained with hematoxylin and eosin (H&E), and examined under a light microscope. The bar is 100 μm. (**B**) Villus height. (**C**) Crypt depth. (**D**) Villus height/crypt depth. Data are presented as means ± standard error of the mean (SEM). * *p* < 0.05; ** *p* < 0.01.

The ileum morphology is shown in Figure 7. Compared with the control group, the mice in the ETEC7 group had a lower average villus height and VH/CD (*p* < 0.05). Nevertheless, treatment with colistin sulfate (COL group), 16 AU rPBD2 (HP group), or 8 AU rPBD2 (MP group) could significantly increase the villus height and VH/CD (*p* < 0.05). However, there were no significant differences among the groups in crypt depth (*p* > 0.05).

**Figure 8:**
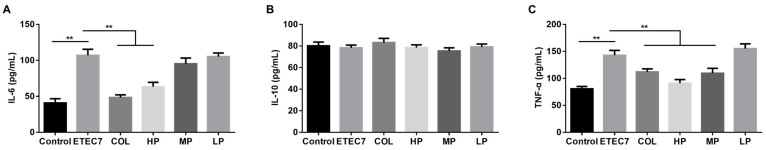
Effect of rPBD2 on serum pro-inflammatory cytokines of ETEC K88-infected mice at 7 dpi. (**A**) Serum IL-6 levels. (**B**) Serum IL-10 levels. (**C**) Serum TNF-α levels. Data are presented as means ± standard error of the mean (SEM). ** *p* < 0.01.

The results of pro-inflammatory cytokine analysis are shown in Figure 8. Compared with the control group, the levels of IL-6 and TNF-α significantly increased in the ETEC group (*p* < 0.05) (Figure 8A,C). Treatment with colistin sulfate (COL group) or 16 AU rPBD2 (HP group) could decrease the levels of IL-6 (*p* < 0.05). Treatment with colistin sulfate (COL group), 16 AU rPBD2 (HP group), or 8 AU rPBD2 (MP group) could also significantly decrease the levels of TNF-α (*p* < 0.05). However, there were no significant differences among the groups in IL-10 levels (*p* > 0.05) (Figure 8B).

## 4. Discussion

Antibiotics are widely used to combat bacterial infection, and sub-therapeutic levels of antibiotics were also widely used as growth promoters in livestock and poultry in the past. However, due to the rapid spread of antibiotic-resistant strains, the use of antibiotics has been strictly regulated, and the use of antibiotics as growth promoters in animal husbandry has been banned in several countries [16]. Therefore, it is urgent to develop safe and efficient antibiotic alternatives to treat bacterial infection. PBD2, a defensin identified from swine, has broad antibacterial, anti-viral, and immunomodulatory activities, and it may be an ideal candidate [36]. There are three strategies to produce PBD2: direct extraction from pigs, chemical synthesis, and heterologous expression [53]. Heterologous expression seems to be an economic method for PBD2 production due to complex processes for extraction and the high cost of chemical synthesis. In one study, His-tagged PBD2 was expressed and purified in *E. coli*, and the purified His-tagged PBD2 had salt-resistance and thermal stability [54]. PBD2 has also been successfully expressed and purified in *P. pastoris* with a yield of 383.7 ± 21.5 mg/L in a 7.5 L bioreactor [34]. Compared to other expression systems, *P. pastoris* has prominent advantages for the expression of AMPs. The advantages include easy genetic manipulation, high-cell-density fermentations, extracellular secretion, and the potential for post-translational modifications [53,55,56]. Furthermore, *P. pastoris* is non-pathogenic, and the cell-free crude supernatant obtained after centrifugation can be used in livestock without further purification [34,53].

In this study, rPBD2 was successfully expressed in *P. pastoris* X-33 using pPICZαA vector. Optimizing the cDNA sequence based on the codon preference of *P. pastoris* may improve the expression efficiency of the target gene. The copy number also affects the expression of the target gene [57,58]. To enhance the expression, PBD2 cDNA was optimized based on the codon usage of *P. pastoris*. Sixty positive transformants were selected, and rPBD2-51 showed the highest inhibition rate to *S. aureus* ATCC 29213. To verify the PBD2 expression, Tricine SDS-PAGE and Western blot were performed, and two clear bands were observed around 6.5 kDa. It has been reported that PBD2 contains three pairs of disulfide bonds, but dithiothreitol may not be able to fully reduce the disulfide bonds of PBD2, thus affecting its migration rate on the polyacrylamide gel.

Changing the *P. pastoris* growth conditions, including the concentration of methanol and the induction time, also affects the expression of recombinant protein [59]. In this study, the optimized induction conditions were 1% methanol for 120 h induction, and the antibacterial activity reached 20 AU/mL.

It has been reported that PBD2 has antibacterial activity against Gram-positive and -negative bacteria [33,34]. In the current research, crude rPBD2 showed a broad spectrum of antibacterial activity against several Gram-positive and -negative bacteria. When AMPs are used in the animal industry, many factors, including temperature, pH, enzymes, and salt ions, may affect their activities. In our study, crude rPBD2 maintained high activity at temperatures up to 60 °C; however, the activity was completely abolished when the temperature was up to 80 °C. This may allow crude rPBD2 to endure the high-temperature granulation process of the feed. Future studies should focus on how to increase thermal stability by site-directed mutagenesis [60]. The pH values of weaned piglets varied from 1.6 to 4.4 in the stomach, 2.4 to 6 in the small intestine, and 5.9 to 6.7 in the large intestine [61]. The gastrointestinal tracts of animals contain gastric enzymes, such as pepsin, and pancreatic enzymes, such as trypsin. The results of pH stability showed that crude rPBD2 maintained high activity in a wide pH ranging from 2 to 10. Additionally, our results showed that crude rPBD2 maintained activity when it was exposed to pepsin and trypsin, which was consistent with previous report [34]. These two results suggested that crude rPBD2 can pass through the stomach and maintain function in the intestine to combat ETEC infection. In addition, salt ions affect the activity of AMPs by decreasing the interactions between AMPs and cell membranes. The antibacterial activity of synthetic human β-defensin 3 (hBD-3) was completely abolished in the presence of 150 mM NaCl or 2.5 mM CaCl_2_ [62]. Veldhuizen et al. [33] also reported that the antibacterial activity of synthetic PBD-2 was completely abolished in the presence of 150 mM NaCl. However, in our study, physiological concentrations of salt ions had little effect on crude rPBD2 activity. We speculate that post-translational modifications in *P. pastoris* enhanced the salt resistance of rPBD2.

Crude rPBD2 was resistant to low pH, proteases, and salt ions, which suggested that it could be used to treat ETEC-induced infection through oral administration. ETEC is one of the most important reasons for PWD in piglets, which causes substantial losses to the pig industry with significant negative impacts on the food production chain [63]. Mice are sensitive to ETEC and are often used to construct ETEC-diarrhea models to evaluate the efficacy of drugs in vivo. In this study, the oral administration of crude rPBD2 alleviated the clinical symptoms, reduced the bacterial colonization in stools and the colon, improved the morphology of the ileum, and decreased the levels of serum pro-inflammatory cytokines in ETEC-infected mice.

ETEC can cause diarrhea and weight loss in mice and pigs [52,63]. Ding et al. [64] reported that MccJ25 decreased the diarrhea scores and body weight losses in ETEC-infected mice. In another report, cecropin AD decreased the diarrhea incidence and increased the body weight gains in ETEC-infected piglets [26]. Similarly to previous reports, crude rPBD2 alleviated the body weight losses, decreased diarrhea scores and the intestinal/carcass ratio, and improved the health of mice. As part of innate immunity, AMPs can inhibit the proliferation of pathogens and maintain host health [65]. Jing et al. [66] reported that LF-6 significantly decreased the loads of *E. coli* in the liver, mesentery, and cecum. In our study, ETEC loads in stools and the colon were significantly decreased after treatment with 16 AU rPBD2. Intestinal morphology, such as villus height and crypt depth, is an important indicator of intestinal health. Villus height, crypt depth, and VH/CD are important metrics for assessing the absorption of the intestine [67,68]. AMPs can efficiently improve the morphology of intestine. Lin et al. [69] reported that antibacterial peptide *Bombyx mori* gloverin A2 (BMGlvA2) improved intestinal morphology by elevating the duodenum villus height and decreasing the crypt depth in the duodenum and ileum in ETEC-infected mice. C-L, a novel hybrid antimicrobial peptide, alleviated the damage to the jejunum and increased the VH/CD in EHEC-infected mice [70]. In the current study, crude rPBD2 prevented the decrease in villus height and VH/CD caused by ETEC. This result indicated that the oral administration of rPBD2 might elevate nutrient absorption within the intestine, which might be the reason why crude rPBD2 had the tendency to prevent the body weight losses caused by ETEC. When a host is infected with ETEC, the expression levels of pro-inflammatory factors, such as IL-6 and TNF-α, will increase [71]. Similarly, in our study, ETEC infection increased the production of IL-6 and TNF-α in mouse serum. It has been reported that AMPs can decrease the levels of pro-inflammatory cytokines in ETEC-infected mice [10,63,69,71]. In agreement with previous studies, the oral administration of crude rPBD2 significantly decreased the production of IL-6 and TNF-α in the sera of ETEC-infected mice. This result indicated that crude rPBD2 could be a negative regulator of pro-inflammatory cytokines and inhibit inflammatory responses.

## 5. Conclusions

In conclusion, PBD2 was successfully expressed in *P. pastoris*, and the activity titer reached 20 AU/mL. Crude rPBD2 had a wide-spectrum antibacterial activity against Gram-positive and -negative bacteria, and high resistance to pH changes, proteases, salts, and temperatures ranging from 20 to 60 °C. Furthermore, crude rPBD2 could alleviate intestinal damage and inflammatory response, and decrease bacterial colonization in stools and the colon in ETEC-infected mice.

## Figures and Tables

**Table 1:** Primers used in this study.

Primer	Sequence (5′–3′)
PBD2F	GCTAAAGAAGAAGGGGTATCTCTCGAGAAAAGAATTAACTTGTTGACTGGTTTG
PBD2R	TCAATGATGATGATGATGATGGTCGACTTATCTGATACAGCACTTGGCCTT
5′AOX1	GACTGGTTCCAATTGACAAGC
3′AOX1	GCAAATGGCATTCTGACATCC

**Table 2:** Antibacterial spectrum of crude rPBD2.

Categories	Genus	Strains	Inhibition Rate (%)
Gram-negative bacteria	*Escherichia*	*E. coli* ETEC7	88.71 ± 0.45
		*E. coli* ETEC17	90.15 ± 0.45
		*E. coli* ETEC20	89.83 ± 0.60
		*E. coli* EPEC28	92.16 ± 0.38
		*E. coli* EPEC48	91.95 ± 0.30
		*E. coli* EPEC66	91.57 ± 0.26
		*E. coli* EPEC133	89.52 ± 0.56
		*E. coli* ATCC 25922	83.03 ± 0.94
		*E. coli* 72	91.15 ± 0.46
		*E. coli* PCN033	90.26 ± 0.64
	*Salmonella*	*S. typhimurium* ATCC 14028	88.46 ± 0.31
		*S. typhimurium* CVCC 542	89.07 ± 0.72
		*S. typhimurium* CVCC 212197	89.10 ± 0.94
		*S. pullorum* C79-13	77.44 ± 0.52
	*Pasteurella*	*P. multocida* 9261	78.48 ± 2.57
		P. multocida HB03	75.44 ± 0.30
	*Actinobacillus*	*A. pleuropneumoniae* 4074	82.84 ± 0.84
Gram-positive bacteria	*Staphylococcus*	*S. aureus* ATCC 29213	88.56 ± 0.54
		*S. aureus* 1213M4A	82.51 ± 1.43
	*Streptococcus*	*S. suis* SC19	77.47 ± 0.72
		*S. suis* 0810	66.31 ± 1.53

## Data Availability

The data for this study can be obtained from the corresponding author upon reasonable request.
